# Using Sensory Wheels to Characterize Consumers’ Perception for Authentication of Taiwan Specialty Teas

**DOI:** 10.3390/foods10040836

**Published:** 2021-04-12

**Authors:** Tsung-Chen Su, Meei-Ju Yang, Hsuan-Han Huang, Chih-Chun Kuo, Liang-Yü Chen

**Affiliations:** 1 Tea Research and Extension Station (TRES), Yangmei, Taoyuan City 326011, Taiwan; tres201@ttes.gov.tw (T.-C.S.); tres608@ttes.gov.tw (M.-J.Y.); a1009@ttes.gov.tw (H.-H.H.); kcc0204@ttes.gov.tw (C.-C.K.); 2 Department of Biotechnology, Mingchuan University, Taoyuan City 333321, Taiwan

**Keywords:** tea competitions, sensory evaluation, labeling, flavor profiling, aroma attribute, traceability

## Abstract

In the context of fair trade and protection of consumer rights, the aim of this study was to combat adulteration, counterfeiting, and fraud in the tea market, and rebuild the image of high-quality Taiwan teas. Experts at the Tea Research and Extension Station, Taiwan (TRES), are engaged in promotion of the systems of origin identification (AOC) and grading for authentication of Taiwan’s premium teas. From tea evaluation competitions (bottom-up quality campaign), the flavor descriptions and consumers’ perceptions were deconvoluted and characterized for the eight Taiwan specialty teas, namely, Bi-Luo-Chun, Wenshan Paochong, High-Mountain Oolong, Dongding Oolong, Tieh-Kuan-Yin, Red Oolong, Oriental Beauty, and Taiwan black tea. Then, according to the manufacturing processes, producing estates and flavor characters, the specialty teas were categorized into six sensory wheels. The flavor descriptors of the sensory wheels were also recognized in consumers’ feedback. In recent years, the performance of international trade in tea also demonstrates that the policy guidelines for authentication of specialty teas are helpful to the production and marketing. Furthermore, the development of sensory wheels of Taiwan’s specialty teas is the cornerstone to the establishment of the Taiwan-tea assortment and grading system (TAGs) for communication with the new generation consumers, enthusiasts, sellers, and producers.

## 1. Introduction

Tea is the most popular beverage in the world after water, and the tea leaf (from *Camellia sinensis*) is also one of the main agricultural commodities in international trade [1]. Taiwan produces many famous tea beverages, due to its unique geographical and climatic environment for tea production, and is influenced by neighboring producers (mainland China, Japan, Korea, Vietnam) within the East Asian tea culture sphere [2,3]. For hundreds of years, the development of tea-related industries has played an important role in Taiwan’s economic and social growth. Today’s tea consumer market has expanded to include merchandise tea, premium tea, bottled tea, and ready-to-drink tea [4]. However, the diversification of culture and international economic activities have caused changes in eating habits that have impacted the industrial supply chain of tea leaves in Taiwan [5]. The topic of tea culture research has attracted new attention.

**Figure 1:**
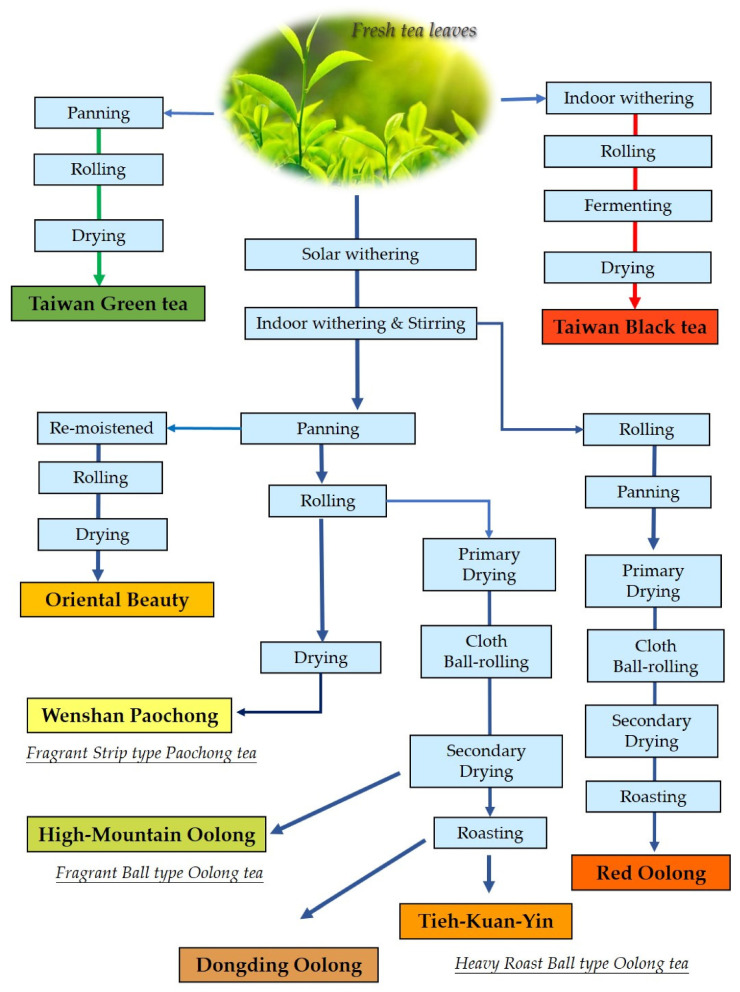
The manufacturing processes and flavor categories of eight specialty teas in Taiwan. The flavors of Dongding Oolong, Tieh-Kuan-Yin, and Red Oolong are assigned to the flavor category of heavy roast ball-type Oolong tea (HRBOT).

Specialty tea consists of whole-leaf or partial loose-leaf tea, in contrast to commodity tea, which is cut and blended for industrial extraction or used in tea bags in stores. As part of the global trend towards healthy eating, tea consumption is related to the phytochemicals in various tea infusions for tastes and physiological activities, which may provide some health benefits for chronic diseases (such as cardiovascular disease and cancer) [6]. Specialty tea is usually expensive, and highly popular due to its unique flavor, excellent qualities, and small supply from tea estates. According to the popularity of the market items, the distinct manufacturing processes of eight Taiwan specialty teas are demonstrated in detail and partitioned into the different flavor categories in Figure 1.

**Figure 2:**
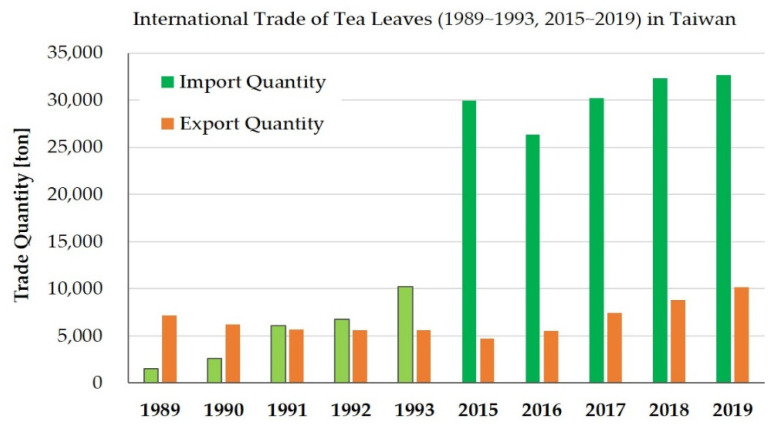
Comparison of import and export trade quantities of Taiwan’s tea-related products in the periods 1989~1993 and 2015~2019. The import and export data were sourced from the agricultural statistics website (accessed on 15 August 2020) of the Council of Agriculture in Taiwan.

Since 1991, the import quantity of Taiwan’s tea has rapidly surpassed the export quantity, as shown in Figure 2. Due to changes in industrial structure, decreased land resources, and rising labor costs, Taiwan has also transformed from a tea exporter to a tea importer to meet domestic consumer demand. The development of the tea industry also emphasizes environmental and sustainable responsibility, and authentication of health, natural, local, and traditional characteristics [7]. Thus, Taiwan’s tea industries have conceived the creation of a high-value, high-quality economic ecology and industrial routes guided by government policies, such as traceability and grading systems in agricultural products [8].

Food authentication is the process of verifying compliance with a food label instruction. Whether from a business perspective or a legal perspective, food authentication has aroused significant interest. Labeling and ingredient regulations may vary by country or region for food safety issues or prevention of criminal activities [9]. Many beverages, such as wine, coffee, and beer, are labeled based on the biological materials [10] and the protected designation of origin and processing methods [11]. However, the genetic bioinformation of fresh tea leaves has been destroyed and is difficult to identify after undergoing processing with physical stress, high temperature drying, and extraction with hot water.

Numerous technological approaches have also been utilized to profile the characteristic fingerprint of beverages with physical or chemical data [12,13]. However, it is difficult to connect these auxiliary verification methods with consumers’ impressions of tea and use them for authentication in tea trade. The Tea Research and Extension Station, Taiwan (TRES) and industrial experts proposed the strategic framework of “Image Shaping of the International Brand of Taiwan Tea and Establishing the AOC System of Taiwan Tea”, in which AOC is an abbreviation of Appellation d’Origine Contrôlée in French, indicating a management system for the origin and nomenclature of agricultural products [14]. The goal of the project is to establish a credible tea label with traceable origin, cultivar, process and flavor information for Taiwanese teas.

Consumers make purchasing decisions based on a variety of quality cues (brand reputation and price) controlled by marketing [15]. The authentication of Taiwan specialty tea is an effective means of improving its production, manufacturing, and marketing. The discriminant descriptions of the origin, variety, appearance, aroma, taste, and manufacturing process of the eight Taiwan specialty teas are listed in Appendix A. In addition to the accurate labeling of the cultivar, origin, processing, and traceability of specialty teas, a number of other key characteristics need to be addressed.

In tea purchases, consumers often need to try individual teas, and determining a favorite flavor is time-consuming. However, consumer preference for a tea flavor is difficult to state, and words used in professional evaluation are too artistic and metaphysical (elegant girly, dame-like, convergent, perfect aging) for others to understand. A recent study noted that the problem stems from the gap between the sensory perceptions of tea beverages by younger consumers and professional opinions in tea marketing [16]. The application of a sensory lexicon will enable consumers to better understand the flavor of tea they purchase and also drive a new fashion in tea tasting, attracting younger generations and more tea lovers [17,18].

With the aim of authenticating Taiwan tea to protect consumer rights and ensure trade fairness, numerous fast and accurate analytical techniques have been developed to combat tea counterfeiting, adulteration, and fraud [19]. The aroma and taste of tea is comprised of complex substances, which are also an important manifestation of tea flavor [20,21]. The sensory wheels provide industry participants with a tool to understand exactly what constitutes the “characteristic taste and aroma” of the Taiwan specialty teas. The lexicon of the sensory wheel could be used as the basis of flavor description to expand knowledge in the field of sensory perception and quantitative description analysis (QDA) of beverages based on formula design, product development, product inspection, and sensory evaluation (competition), in addition to consumer science [22,23,24].

This aim of this study was to establish a systematic sensory lexicon to certify the flavor characteristics of specialty teas with high-quality, as flavor authentication. As the major official institute involved in the promotion of the tea industry in Taiwan, TRES must coordinate manufacturers, tea traders, and consumers to establish a functional medium with well-certified guidelines to reduce the gaps in traditional tea culture. Using an expert taster who evaluated the appearance, color, smell, taste, and mouthfeel, the overall impression of the quality of the tea infusion was obtained with a balance based on defect-free and sensory properties. Furthermore, the official sensory wheels of Taiwan specialty teas were developed for the tea industry to serve as communication and quality control tools to enhance the marketing of Taiwan tea for consumers, and to strengthen the core competitiveness of tea farmers. In this study, the characteristic flavors of Taiwan specialty teas were clearly identified based on consumers’ perception using the flavor attributes of the sensory wheel.

## 2. Materials and Methods

### 2.1. Experimental Design and a Focus Group

The main structure of this research consists of the eight specialty teas and six categories of sensory wheels, which have the flavor attributes of infusion color, taste, and aroma in the consumers’ perception. Since the inception of TRES, the lexicon of flavor perceptions on teas has been dynamically reviewed by the professional staff. Our study uses the concept of identifying tea flavors to develop a sensory wheel with flavor terms in detail, and then verifies the accuracy of the flavor terms in each sensory wheel associated with the Taiwan specialty teas.

Based on the sensory wheel, tea traceability establishes flavor clues in authentication, and can be used to express the flavor and quality perceptions of Taiwan specialty tea as a marketing tool. The official sensory wheels can be promoted to participating tea merchants, producers, and tea farmers to understand the basic quality requirements and flavor items in the evaluation. The protocols, including sensory evaluations and consumer surveys in this study, are certified as an exempted approval (document number: 1103408809) by the institutional review of TRES according to Article 5 of the Human Subjects Research Act (2 January 2019) of the Ministry of Health and Welfare, Taiwan. All identified data have been delinked for participants’ anonymity and confidentiality before analysis.

### 2.2. Sensory Evaluation Panel

The flavor wheel working group and the sensory evaluation panel are composed of 25 professional researchers engaged in tea manufacturing and tea sensory evaluation at TRES, Taiwan. The panelists were recruited from certificated referees with qualifications obtained from training courses and practice assessments conducted by TRES for top-level tea sensory appraisers. The certification system of tea sensory appraisers in Taiwan is developed and classified in five levels, namely, elementary, junior, senior, advanced, and special appraisers. Certified appraisers must obtain the qualifications of appropriate training courses and assessments for tea sensory evaluation at different levels, consistent with the ISO 8586:2012 guidelines for sensory analysis [25]. By gender and age, the membership consists of 7 males and 7 females in the 20–40 age group, 4 males and 2 females in the 40–60 age group, and 1 male and 1 female in the 60–65 age group. The function of the focus group was to determine the attractive, significant, and associated attributes of flavors from the premium specialty teas in tea evaluation competitions rather than teas that are more easily available in stores.

### 2.3. Collection of Tea Samples

The development of the tea sensory wheel was used to standardize the flavor description and sensory evaluation of premium tea, so the high-quality tea samples were collected from tea evaluation competitions conducted from 2019 to 2020 in Taiwan. The flavor attributes of Taiwan green tea were assessed and graded using 40 samples of Bi-Luo-Chun green tea. The flavor attributes of the fragrant strip-type Paochong tea were assessed and graded using 50 samples of Wenshan-Paochong tea. The flavor attributes of the fragrant ball-type Oolong tea were assessed and graded using 73 samples of High-Mountain Oolong produced in locations including Nantou, Chiayi. The flavor attributes of the heavy roast ball-type Oolong tea were assessed and graded using a total of 210 samples of Dongding Oolong (169 in Nautou), Tieh-Kuan-Yin (19 in Muzha), and Red Oolong (22 in Taidong). The flavor attributes of Oriental Beauty tea were assessed and graded using 62 samples. The flavor attributes of Taiwan black tea were assessed and graded using a total of 60 samples of Sun-Moon-Lake black tea (20 in Nautou), Small-leaf black tea (20), and Honey-flavor black tea (20 in Hualien).

### 2.4. Preparation of Tea Infusions and Sensory Evaluation

The tea infusions were prepared as a liquor extraction with dry tea leaves, as described in the international standard ISO 3103:1980 [26]. The tea brewing method was to weigh 3 g (with an accuracy of ±2%) of tea samples into the cup set (pot and lid) specified by ISO 3103:1980, then pour a quantity of 100 °C boiled water equal to 50 times the tea leaf weight for the specified brewing time (listed in Table 1). The tea infusion was transferred to an inspection bowl, before waiting for 6 to 8 min for the tea infusion to cool to 45 °C prior to tasting by the sensory panelists.

Results of sensory evaluation in terms of color, flavor, taste, and aroma were conducted with hedonic scaling and a ranking test to determine a suitable grade based on the flavor attributes of the tea samples.

### 2.5. Survey of Consumers’ Acceptance of Aroma Descriptors in the Sensory Wheels

To explore the feedback of consumers regarding the official sensory wheels of Taiwan specialty teas, participants were randomly invited to participate in a questionnaire survey at the Taiwan International Tea Expo on 11–16 November 2020 at the Taipei Nangang Exhibition Center. Participation was voluntary; participants had given verbal informed consent before the trial and could withdraw freely at any stage. A total of 90 valid questionnaire responses were obtained, including those of four foreigners with a questionnaire in English. Of the total number of respondents, 61% were male and the median age was 45 years old. Although the sensory wheel has different color attributes and the same taste attributes of tea infusions, it is difficult to analyze using questionnaires. Therefore, this questionnaire analyzed a total of 25 aroma attributes in six sensory wheels to profile the aroma characteristics of various Taiwan specialty teas. The design of the questionnaire used the Check-All-That-Apply method to verify the recognition coverage of consumer perception of the flavor attributes of Taiwan specialty teas.

Based on the questionnaire data, the recognition percentage of an aroma attribute according to a consumer’s perception was calculated and transformed into a 5-point scale, named the acceptance index of aroma attribute. For an aroma attribute with a recognition percentage of 45% or more, 5 points were given, 44.4–35% were given 4 points, 34.4–25% were given 3 points, 24.4–15% were given 2 points, and 14.4–1% were given 1.1 points. Those not recognized are not presented.

## 3. Results and Discussion

### 3.1. Authentication and Sensometrics of Eight Taiwan Specialty Teas

**Figure 3:**
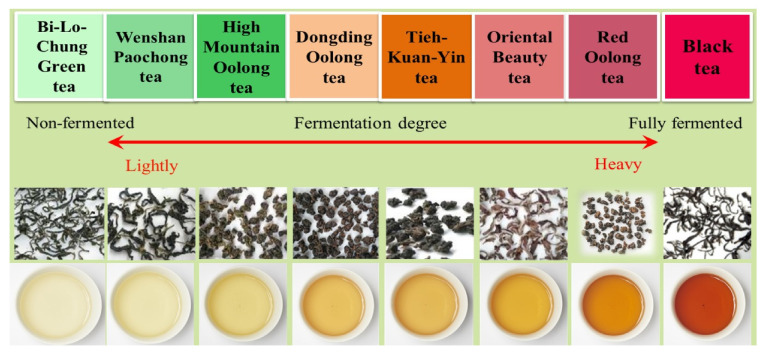
The eight specialty teas in Taiwan were characterized by their fermentation degree, shape, and infusion color.

Based on the production estates, the unique manufacturing process, and consumer impressions of the premium teas, the TRES focus group selected eight Taiwan specialty teas to characterize their flavor attributes. According to the tea manufacturing process, the color of tea infusion deepens with the degree of fermentation, as shown in Figure 3.

Taiwan is the main producing area of partially fermented Oolong tea in the world [27], so the Oolong teas are more diverse, and the flavor spectrum is broader than that of green or black tea. Oriental Beauty tea is famous for its organic production and unique honey flavor, and is infested by the common tea insect (*Jacobiasca formosana*), which feeds on leaves, stems, and buds without pesticide residues. The tea plant induces the secretion of the monoterpenediol and hotrienol, which gives the tea its unique flavor [28]. Because of Oriental Beauty’s unique method of planting and processing of the tea raw material, it is also called White-tip Oolong tea [29].

Green tea is an unfermented tea made with relatively low processing, but differences in processing nonetheless have a significant impact on the flavor (mainly aroma) and appearance [30]. Based on its different manufacturing processes, green tea is generally classified into four sub-categories, namely roasted (pan-fired) green tea, steamed green tea, baked green tea, and sun-dried green tea. A recent study showed a significant difference in the composition and content of pyrazines, because the aroma tracking molecule is different between flat and strip green teas [31].

According to the basic processes of tea manufacturing, as shown in Figure 1, regardless of the variety, tea leaves from cultivated varieties can be made into various teas. However, the flavor suitability must be considered. Following long-term practical experience and marketing verification, tea farmers know that large-leaf cultivars (C. *sinensis* var. *assamica*) with a high level of catecholamine are suitable for the flavor of black tea, and small-leaf cultivars (C. *sinensis* var. *sinensis*) with a low level of catecholamine are suitable for the flavors of green tea and partially fermented teas [32]. Due to the importance of suitability [33], green tea is not made with bitter large-leaf varieties, and the quality is not as high as that of small-leaf cultivars.

From the perspective of the producing estate, well-managed tea trees can be harvested for at least 30 to 40 years, and specific varieties are also suitable for planting and growing in the producing area of the specialty tea. Thus, the regeneration of tea planting varieties is very slow [34]. However, improved processing techniques using appropriate tea materials were used to produce uniquely flavored tea in non-traditional locations, causing consumers to mistake the authentication and identification of tea.

Taiwan black tea is dominated by large-leaf cultivars. Characteristic Honey-flavor black tea is predominantly produced at the Hualien estate and made from Large-leaf Oolong, which is a tea tree of a small-leaf cultivar. Production of partially fermented teas, such as Paochong tea or Oolong tea, uses common small-leaf cultivars of Chin-Shin-Oolong and TTES No. 12 (Jhinhsuan). Because the catechin content of tea leaves in summer is too high and the flavor is too bitter for partially fermented teas [33], ten years ago tea farmers in western Taiwan converted the summer tea leaves of small-leaf cultivars into black tea, in line with TRES’s suggestions.

### 3.2. Sensory Wheels to Characterize the Six Flavor Categories in Taiwan Teas

**Figure 4:**
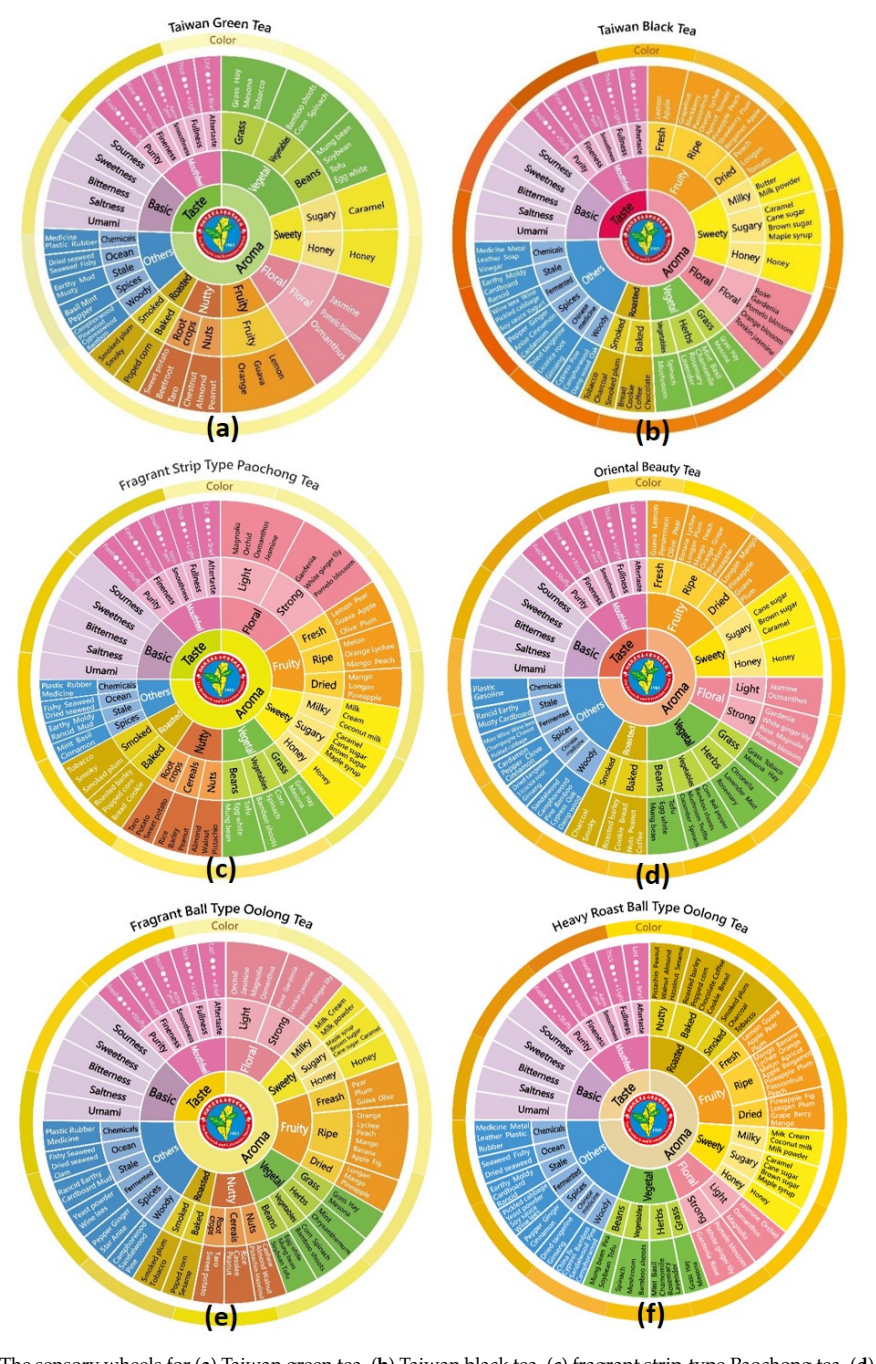
The sensory wheels for (**a**) Taiwan green tea, (**b**) Taiwan black tea, (**c**) fragrant strip-type Paochong tea, (**d**) Oriental Beauty tea, (**e**) fragrant ball-type Oolong tea, and (**f**) heavy roast ball-type Oolong tea. The high-resolution graphs are reproduced with copyright permission from the Tea Research and Extension Station (TRES), Taiwan (https://www.tres.gov.tw/ws.php?id=3744, accessed on 15 April 2020).

Flavor affects the acceptance and choice of food intake, and helps people identify potentially harmful compounds. Based on the unique flavor attributes and consumer impressions, the sensory wheel working group further developed six sensory wheels of the flavor categories of specialty teas, which are Taiwan green tea (TGT), the fragrant strip-type Paochong tea (FSPCT), the fragrant ball-type Oolong tea (FBOT), the heavy roast ball-type Oolong tea (HRBOT), Oriental Beauty tea (OBT), and Taiwan black tea (TBT), as shown in Figure 4a–f.

The official sensory wheel has the function of setting standards and guidelines for each of Taiwan’s specialty teas for the development of Taiwan tea industries, consumer behavior, and trade. The design framework of the sensory wheel has distinctive appearance, context, flavor lexicon, and sensory information, and includes the infusion color and an image representing the tea. Sun-Moon-Lake black tea, Small-leaf black tea, and Honey-flavor black tea are the three representative teas of TGT in Figure 4b. Dongding Oolong tea, Tieh-Kuan-Yin tea, and Red Oolong tea are the three representative teas of HRBOT in Figure 4f. The images of representative tea were displayed on the reverse of the respective sensory wheel, but are not shown here.

Flavor studies [35,36] utilizing chemometric technologies combined with sensory description and consumer perception can provide more reliable criteria for authentication of Taiwan specialty teas. Establishment of easily accessible and stable reference materials is a topic that requires urgent follow-up research. Descriptive sensory analysis, gas chromatography coupled with mass spectrometry (GC–MS), and GC–olfactometry (GC-O) have been used to profile the herbal flavors of different origins and processing methods [37]. Identification of vegetable-like off-odors in vanilla sugar was found to result from microbiological contamination [38]. Excellent chocolates of different brands have been profiled using descriptive sensory analysis [39].

Flavor profiling identifies individual sensory attributes that contribute to the overall sensory impression of the tea infusion and evaluates its strength to establish a description of the flavor and aftertaste, such as in the case of mouthfeel, shown in Figure 4a–f. Therefore, flavor profiling combined with advanced chemical analysis technology can provide reliable recognition and identification tools for agricultural products and food [40]. If the chemical nature of a flavor compound (including off-flavor) is identified, it can be applied to consequential flavor enhancement and quality stabilization. Flavor profiling is not only essential for off-flavor detection, but also if the product is considered to be outstanding. Moreover, marketing of specific processes, raw materials, or storage could result in an improvement of tea production. Illegal activities in the form of adulteration, low quality products, pesticide residues, and forged origin, which may even have harmful health effects, will continue. Therefore, promoting the authentication of premium or specialty teas is a necessary and urgent issue for the Taiwanese tea industry.

### 3.3. Standardizations on Tea Evaluation with Sensory Wheels

The TRES is an official professional institution providing counseling for tea industry development and plays the role of guidance and supervision in the marketing of Taiwanese tea. However, flavor descriptions and standards of Taiwan teas are currently lacking a means of authentication compared to the international classification standards [27,37] of Chinese tea promoted by mainland China. For example, mainland China promulgated national standards, GB/T 39563-2020 and GB/T 39562-2020, for Taiwanese Oolong tea and its manufacturing specifications, respectively.

The official sensory wheel can be used not only as the flavor standard of the Taiwan specialty tea and an auxiliary tool for marketing, but also as teaching material for personnel training and the dissemination of tea-related knowledge on sensory evaluation. The six sensory wheels standardize the flavor lexicon for aroma and taste attributes, and provide a color atlas as a visual reference, as listed in Table 2.

From the comparison of the flavor components of the two extremes in Table 2, the aroma descriptors of TBT are more than those of TGT, which are dominated by a fruity aroma with an absence of a nutty aroma. The ripe fruity aroma attribute is increased (2 to 15), but the nutty aroma attribute is decreased (6 to 0) with the fermentation. The roasted aroma of HRBOT is characteristic and synchronized with fruity, vegetable, and woody characteristics in the roasting process. The roasted nutty aroma is a unique characteristic of the Taiwan specialty teas of HRBOT. The process and appearance of OBT are similar to those of FSPCT, but the dissimilarity in the raw materials of tea leaf and the fermentation degree causes significant differences in the nutty, woody, and herbs aroma attributes. The saltiness of the basic taste is not commonly perceived in tea infusion evaluations, and the distinction between astringency and bitterness should be more refined [41].

In the future, the main challenge of psychological measurement will be to find a scale with proportional characteristics that is not affected by other factors used in the surrounding environment [15]. Thus, an ideal sensory scale infers that the scale should have a well-defined reference point [16] and a clean baseline, the psychological distances between units on the scale should be the same, and the scale will provide the same outcome regardless of any disturbance of environmental factors, such as temperature, light, or other stimuli.

### 3.4. Aroma Profiling of Taiwan Specialty Teas by Consumers’ Perception with the Sensory Wheels

Flavor profiling includes formal procedures to describe and evaluate the taste, smell, mouthfeel, and flavor, in addition to an underlying complex of sensory impressions of a product in a reproducible manner. Taste is the sensation experienced by taste receptor cells located on the taste buds when stimulated by certain soluble substances. The sense of taste is combined with the olfactory and trigeminal system to produce an overall flavor [41]. However, aroma development is the most abundant and complex item in the sensory evaluation of tea infusions.

**Figure 5:**
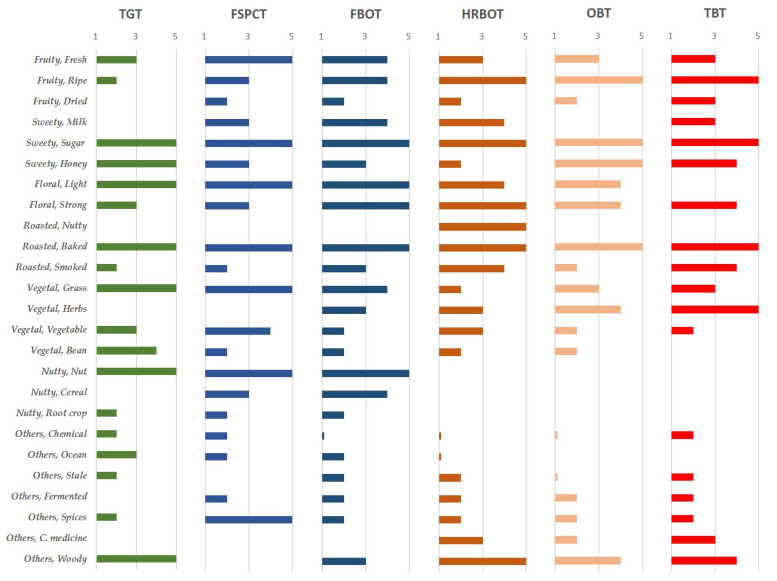
Profiling the acceptance index of the consumers’ sensory perception of the aroma attributes of each flavor wheel. A 5-point scale is used for the acceptance index of the horizontal axis.

According to the results of the consumer questionnaire, the aroma descriptors in the sensory wheels were used to profile the characteristics of Taiwan specialty teas by fuzzy algorithm [42,43], as shown in Figure 5. Apart from the fully fermented black tea and Bi-Lo-Chung green tea in the northern estates, the teas are partially fermented Oolong teas. The flavor characteristics of green and black teas have also been widely accepted by consumers [44,45]. The profiling of the acceptance index of consumers’ sensory perception of aroma attributes can significantly distinguish various teas by the ripe fruity, nutty, or herbs vegetal attributes with the different degrees of fermentation for classification. Spices others and woody others can be used to describe the special aroma attributes for the fragrant strip-type Paochong tea (FSPCT) and the Taiwan green tea (TGT), respectively. Otherwise, the sugar sweet, floral, and baked roasted attributes are the characteristic aromas of Taiwan specialty teas.

Sensory evaluation techniques of QDA [46], consumer testing [44], and hedonic scaling will be used to establish an evaluation system and characterize the most important flavor in Taiwan tea that is widely recognized all over the world. A sampling bias would be raised by the small size of the respondents for a consumer survey on the sensory wheels (2020) during the COVID-19 pandemic, which would reduce the likelihood of receiving this questionnaire. Thus, the data of consumers’ perceptions were not grouped, they only served as the supporting evidence for the effectiveness of flavor authentication.

### 3.5. Development of Sensory Wheels to Enhance the Tea Economics

**Figure 6:**
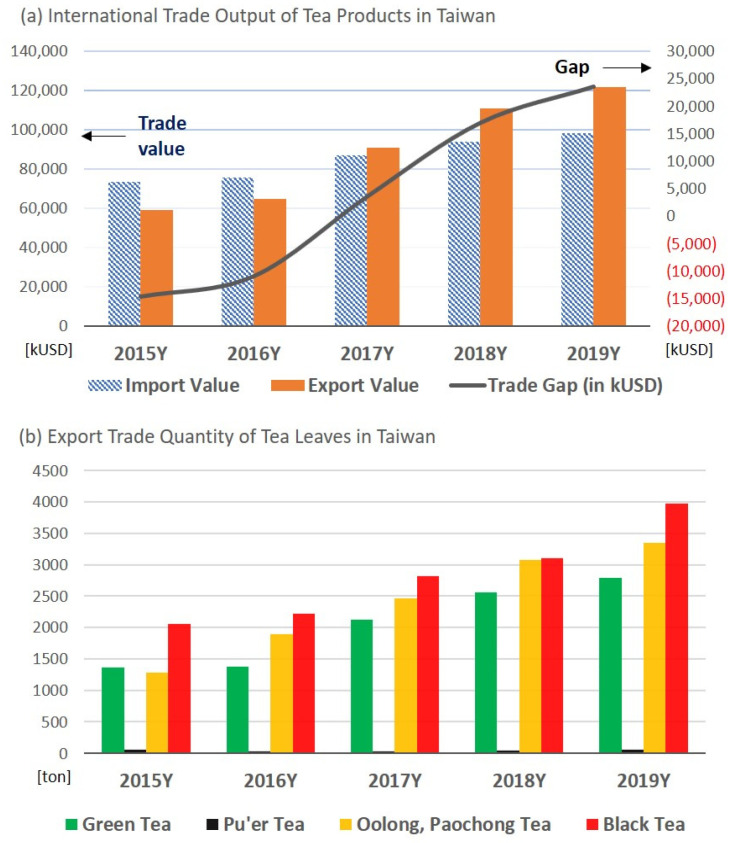
The international trade of Taiwan tea: (**a**) values of foreign exports, imports, and gap (red numbers show the deficit values) for 2015–2019; and (**b**) quantity of four sub-items of foreign exports. The import and export volumes of tea are based on pure tea leaves, and exclude tea extracts or related products. The data source is the tea industry trade union in Taiwan (2020).

Figure 6a shows that Taiwan’s import and export values of tea-related commodities resulted in an export surplus in the trade balance after 2017. Globally, the top three tea exporters were China, India, and Kenya in 2018. China’s export varieties are mainly green tea, accounting for 70% of global green tea exports. However, China’s tea imports are mainly black tea, with Sri Lanka and Taiwan being the top two sources [47]. This result demonstrates that Taiwan’s tea export market still has a considerable economic scale.

The development of a variety of tea processed products has changed the culture of people drinking tea. In particular, the product concept of ready-to-drink tea has created a new tea culture [4]. Tea is no longer limited to freshly brewed hot tea but can also be a refreshing beverage. The change in tea consumption patterns is not only underway in Taiwan, but also other countries. Taiwan’s tea imports are increasing steadily, as shown in Figure 1, because tariffs have been reduced, and domestic tea demand has increased and cannot be met by domestic production.

As shown in Figure 2, the quantity of tea imported by Taiwan is about three times the quantity of tea exported. A further review is needed to determine how Taiwan’s exports of tea can create greater net trade profit. Taiwan exports mainly black tea, semi-fermented Oolong tea, green tea, and a small quantity of post-fermented Pu’er tea, as shown in Figure 6b. Semi-fermented Oolong teas have shown the fastest growth rate during the past five years, which has been higher than those of black and green teas. Taiwan tea imports are cheaper raw materials or products for domestic use or processing, whereas exports are higher-priced tea products, thus resulting in a surplus in the tea trade. The image of high-quality Taiwan tea has been clearly recognized by international tea consumers.

### 3.6. Limitations of this Study and Future Applications

Nevertheless, this study is limited to specific areas and objects and cannot be accurately projected into the larger commercial tea or beverage markets. The quantitative data of flavor description are regarded as representing the objectivity and scientific statement of sensory evaluation [8]. The median age of the questionnaire respondents was 45 years old. The age distribution of consumers who prefer specialty teas is older than that of general tea beverage consumers (20–35 years old). As a sampling bias, the authors consider that older consumers have a better social and economic capacity to purchase specialty teas. Many attributes and characteristic words must consider the consumer’s perspective to achieve the same level of cognition. Through the standard samples of Taiwan specialty teas and the training of sensory evaluation for experts and consumers, the systematic deviations of flavor evaluation should be effectively decreased. The probability of the bias-deviation tradeoff also could be reduced by the comparison with normalized patterns.

For a long time, tea has been an important daily drink for East Asians. Drinking tea will make people feel happy because of its unique aroma, flavor, and taste. However, the sensory wheels are constructed with the criteria that were proposed by professionals for premium Taiwanese teas, and might not accurately project the consumer’s perception of teas in general markets, such as the aroma attributes of root crop-nutty and bean-vegetal, which have lower acceptance in the consumer survey in Figure 5. Because of flavor perceptions related to consumers’ life experiences, the flavor of black nightshade or wet bamboo is difficult to distinguish by people in cold zones, which is not conducive to foreign trade. The sensory wheel requires additions, removals, and dynamic adjustments to reduce the intergenerational and geographic differences in various markets [48]. TRES will invite consumers and experts to evaluate the tea samples of Taiwan to confirm the use frequency of flavor words and quantitative sensory scaling.

From the perspective of the industrial development of beer, wine, coffee, and other popular beverages, professional knowledge education, product contests, media propagation, cultural links, and studies of health benefits can expand the penetration of tea beverages in society [13]. The first step is the involvement of a greater number of people to promote the premium tea marketing. The official sensory wheels can be used as a basic training material in the Taiwan-tea Assortment and Grading system (TAGs) to overcome several obstacles in the sensory evaluation of tea, and to improve the understanding and having contact with high-quality teas.

## 4. Conclusions

In summary, the development of premium teas has gradually dominated the international trade trend and supply chain structure of the entire tea industry in Taiwan. However, the expansion of the supply chains and the diversification of products have led to the prevalence of illegal business practices in the tea industry. Consumers’ impression and perception have gradually faded for the high-quality Taiwan teas. Therefore, promoting the authentication of specialty teas is an urgent issue for the tea industry. Due to the generational differences of food culture and the rise of international industrial specialization, the flavor information of tea products must be dynamically updated and adjusted for the consumer groups whose lifestyle changes.

The implementation of flavor authentication can be used to prevent the forgery of labeling in a traditional certification system of beverages, and the flavor lexicon can also expand consumers’ interest in premium tea via assessor training for sensory evaluation. Further, the analytical techniques should be developed to establish an accurate and quantitative evaluation about the tea flavor. If the scientific clues of a flavor compound are identified, it can be applied to discriminate the quality, origin, and harmful residues of tea. The flavor profiling of the sensory wheels can be applied on the identification of specialty tea with Taiwanese label from general markets. The gap between industrial standards and consumer demand could be repaired with the development of sensory evaluation on tea flavors. Therefore, the use of quantitative descriptions of sensory evaluation, chemical analysis technology, and consumer testing to characterize the most important flavors of Taiwan specialty teas will help establish flavor standards, product traceability, and responsibility.

## Figures and Tables

**Table 1:** The brewing time and flavor categories applied for the eight specialty teas.

Specialty Tea	Samples	Brewing Time	Flavor Category
Bi-Luo-Chun	40	5 min	Taiwan green tea
Wenshan-Paochong	50	5 min	fragrant strip-type Paochong tea
High-Mountain Oolong	73	6 min	fragrant ball-type Oolong tea
Dongding Oolong	169	6 min	heavy roast ball-type Oolong tea
Tieh-Kuan-Yin	19	6 min	heavy roast ball-type Oolong tea
Red Oolong	22	4 min	heavy roast ball-type Oolong tea
Oriental Beauty	62	5 min 30 s	Oriental Beauty tea
Black tea	60	5 min	Taiwan black tea

**Table 2:** Development of the lexicons used in six sensory wheels to characterize the flavor perceptions of experts and consumers for Taiwan specialty tea infusions.

	TGT ^1^	FSPCT ^1^	FBOT ^1^	HRBOT ^1^	OBT ^1^	TBT ^1^
**Color Attribute**						
Color atlas of infusion	10	11	12	14	16	12
**Taste Attribute**						
Basic (Sour, Sweet, Bitter, Salt, Umami)	5	5	5	5	5	5
Mouthfeel (Purity, Fine, Smooth, Full, Aftertaste)	5	5	5	5	5	5
**Aroma Attribute**						
Fruity	4	16	18	26	21	21
(Fresh, Ripe, Dried)	(2, 2, 0)	(7, 6, 3)	(5, 9, 4)	(5, 13, 8)	(5, 11, 5)	(2, 15, 4)
Sweet	3	10	8	10	4	8
(Milk, Sugar, Honey)	(0, 2, 1)	(3, 6, 1)	(3, 4, 1)	(4, 5, 1)	(0, 3, 1)	(2, 5, 1)
Floral	3	8	11	12	8	5
(Light, Strong)	(2, 1)	(4, 4)	(4, 7)	(5, 7)	(3, 5)	(-, 5)
Roasted (Nutty, Baked, Smoked)	3	9	7	23	9	8
	(-, 1, 2)	(-, 6, 3)	(-, 4, 3)	(7, 9, 7)	(-, 7, 2)	(-, 4, 4)
Vegetal (Grass, Herbs, Vegetable, Bean)	12	13	15	22	22	12
	(3, -, 5, 4)	(3, -, 6, 4)	(4, 2, 5, 4)	(3, 6, 8, 5)	(6, 6, 7, 3)	(3, 5, 4, -)
Nutty (Nut, Cereal, Root crop)	6	9	8	0	0	0
	(3, -, 3)	(3, 3, 3)	(5, 3, 2)	(-, -, -)	(-, -, -)	(-, -, -)
Others						
Chemical	3	3	3	5	2	5
Ocean	3	3	5	3	-	-
Stale	3	4	4	4	4	4
Fermented	-	-	2	4	6	5
Spices	3	3	3	3	4	5
Chinese medicine	-	-	-	2	3	4
Woody	4	-	3	6	7	5
**Aroma descriptors ^2^**	58	87	99	131	100	92

^1^ The abbreviations are described as the same as the six sensory wheels in Section 3.2. ^2^ Aroma descriptors are the sum of all aroma attributes, including fruity, sweet, floral, baked, vegetal, nutty, and others.

## Data Availability

Data available within the article.

## Appendix A

The information regarding product traceability (in terms of representative items, cultivar, processing, and producing estate) and the general descriptions of flavor attributes with sensory perceptions are listed for the eight Taiwan specialty teas available in the market.

1. Taiwan Green tea (TGT) is a non-fermented tea. Panning or steaming is the first processing step without withering or fermentation. Bi-Lo-Chung is a representative of the pan-fired green teas and is produced in Sanshiar of New Taipei City. The appearance of Bi-Lo-Chung tea is fresh green, and it is covered with more leaf pubescence, and curled into a tight and spiral shape. The infusion color is pale yellow-verdancy. It possesses a slightly vegetable incense, sweet, and mellow taste. Long-Chin tea looks like sword-blades. The infusion color is pale yellow-green. It has a fragrant aroma, sweet taste, lively and zippy flavor, and refreshing taste. Their qualities also focus on the fresh activity.
2. Oriental Beauty tea (OBT) is academically known as White tip Oolong tea, also known as Penfong tea. It is one of the most fermented teas in the partially fermented tea category. The raw materials are plucked from young and tender tea buds whose stylets were sucked by the small green leafhopper. After withering, the fermentation of the tea leaves is controlled by manual stirring to give the teas their unique honey flavor or mature fruit taste. In this kind of tea, 50%–60% of the catechins in tea leaves are oxidized. It is a unique tea that was locally researched, developed, and made in Taiwan. The tea-making process of this kind of tea includes heavy withering and heavy stirring; after pan firing, the tea leaves must be stuffed and wrapped in a damp cloth to remoisten them; then, they are rolled into shape. It is mainly produced in the tea regions including Shiding, Longtan, Beipu, Er-mei, Toufen, and Tongluo of Miaoli. The made-tea appearance alternates between white, green, yellow, red, and brown colors, like a flower. The top-grade teas have a white tip; tea liquor shows an orange red color, has a unique honey flavor or mature fruit taste, and its taste is supple and mellow.
3. Wenshan Poachong (WSPC) is a partially fermented tea with less fermentation. The main production areas are Nangang, Wenshan in Taipei City and Pinglin, Shiding, Shenkeng, Xizhi in New Taipei City. Its natural appearance is curved in strips and the color is dark green. The infusion color is honey-green and yellow, the fragrance is elegant like a flower, and the taste is sweet and fresh. Its reputation is based on its fragrance and refreshing taste. The flavor category is classified as the fragrant strip-type Paochong tea (FSPCT).
4. High-Mountain Oolong is a partially fermented tea. The major producing estates of Taiwan High-Mountain Oolong are in high altitude areas (altitude 1000 m or above) of Fushin, Hopin, Chia-I, and Nantou counties. These high mountain areas are cold with cloudy mist at the canopy lasting throughout the day. The average sunshine period is also short. Tea leaves and buds of High-Mountain Oolong are tender. The mesophyll is thick and pectin content is high. The bitter and astringency content is low, and the sweet content increases in tea infusions. Hence, fragrant high-mountain teas have characteristics, such as a verdant appearance, honey-green or honey-yellow liquor color, sweet taste, refined aroma, and enduring brewing. High-Mountain Oolong is a Taiwan tea which possesses both a unique aroma and taste. The flavor category is classified into the fragrant ball-type Oolong tea (FBOT).
5. Dongding Oolong is a medium roasting degree type Oolong tea, and is a partially fermented tea with medium fermentation, medium roast, and semi-spherical or spherical shape. Dongding Tea was originally produced in the Lugu mountain area of Nantou (500–800 m above sea level), and its manufacturing technology has been extended to Zhushan, Mingjian of Nantou, and other tea estates in Taiwan. Due to its heavy roasting, Oolong tea must pass through cloth ball rolling and roasting processes, so its appearance is a tightly formed semi-spherical or spherical shape. The appearance color is dark green and oily, the liquid color is golden and bright, the fragrance is elegant and rich, the taste is mellow and sweet, and after drinking the aftertaste lingers. The flavor category is classified into the heavy roast ball-type Oolong tea (HRBOT).
6. Tieh-Kuan-Yin also is a partially fermented tea with medium fermentation, heavy roasting, and semi spherical or spherical shape. At present, the manufacturing method is similar to that of semi-spherical Oolong tea. After the tea leaves are withered, the fermentation process is controlled by adjusting the stirring process to increase their fermentation degree; the teas are then repeatedly roasted to increase their roasting degree, so the components in the teas are transformed into a unique fragrance and flavor by the temperature of the fire. The top grade Tieh-Kuan-Yin tea is made from the Tieh-Kuan-Yin cultivar, which is mainly produced in Muzha and Yìng-Jhih-Hongsīn cultivar in Shimen. The increase in roasting degree by repeated roasting transforms the components in the teas into a unique fragrance and flavor due to the fire temperature. Therefore, after brewing, the tea infusion color is amber and slightly red, the taste is rich and mellow, lightly astringent although sweet and smooth, with a pure and weak fruit sour flavor. After repeated brewing, a fragrant and mellow aftertaste is preferred. The flavor category is classified into the heavy roast ball-type Oolong tea (HRBOT).
7. Red Oolong is a partially fermented tea with moderate fermentation. Red Oolong is a newly created tea that combines the processing characteristics of Oolong tea and black tea. It has the highest degree of fermentation among Oolong teas currently available. The appearance of Red Oolong is hemispherical and dark red with luster. The color of the tea infusion is amber, orange, and red like black tea, but the taste is an Oolong tea flavor. It has a distinct floral, fruit, or honey aroma, a sweet and smooth taste, and is a prevailing specialty tea suitable for hot or cold brewing. The flavor category is classified into the heavy roast ball-type Oolong tea (HRBOT).
8. Taiwan Black tea (TBT) belongs to the fully fermented tea category, which is made from tea leaves after withering, rolling, and fermenting. Black teas made from the large-leaf cultivars, such as TTES No. 8, No. 18, and No. 21 produced mainly in Yuchi and Puli tea estates in Nantou county, have a special tea flavor and high quality. Honey-flavor black tea is a specialty tea produced in Hualien and Taitung (eastern Taiwan). In recent years, other tea estates have also used tea leaves of summer and autumn small-leaf cultivars as raw materials to produce black tea. Taiwan black teas are popular among consumers. The appearance and color luster of black tea is preferred to be black, oily, and moist, and showing a purple light. The leaf twists are tight, uniform and neatly knotted, and preferred to have golden white fuzz (except TTES No. 18). The color of tea liquid is red and glamorous, clear and bright, and displays a luminosity. The aromas of black teas produced from different cultivars have their own features, but all are preferred to be pure and rich. The taste is mellow, thick, and strong, and fresh and refreshing. Infused tea leaves are soft and lively, and have a uniform bright red color.
